# Performance comparison of artificial intelligence models in predicting 72-h emergency department unscheduled return visits

**DOI:** 10.3389/fpubh.2025.1609206

**Published:** 2025-12-19

**Authors:** Lumin Fan, Xinghua Zuo, Lunxian Tang, Honglin Xiong, Yanli You, Chongjun Fan

**Affiliations:** 1 Business School, University of Shanghai for Science and Technology, Shanghai, China; 2 Asset Management Department, Shanghai East Hospital, School of Medicine, Tongji University, Shanghai, China; 3 Department of Emergency Internal Medicine, Shanghai East Hospital, School of Medicine, Tongji University, Shanghai, China; 4 Collaborative Innovation Center for Biomedicine, Shanghai University of Medicine & Health Sciences, Shanghai, China; 5 Faculty of Medicine, University of Banja Luka, Banja Luka, Bosnia and Herzegovina; 6 Shanghai Jingan District College (Shanghai Open University Jingan Branch), Shanghai, China

**Keywords:** emergency department, unscheduled return visits, machine learning, deep learning, comparative assessment

## Abstract

**Background:**

Unscheduled return visits (URVs) to emergency departments (EDs) contribute significantly to healthcare burden through resource utilization and ED overcrowding. While artificial intelligence (AI) methodologies show potential in URV prediction, existing studies have employed limited algorithms with moderate performance, highlighting the need for comprehensive AI architecture comparison within unified cohorts.

**Objective:**

This study evaluated the predictive performance of multiple AI models for 72-h ED URVs, aiming to identify optimal risk stratification strategies for improved discharge planning and targeted interventions.

**Methods:**

This retrospective study analyzed adult internal medicine visits to the ED at a tertiary hospital. URVs were defined as ED revisits occurring within 72 h after initial ED discharge time. The dataset was partitioned into training (70%) and testing (30%) sets. Four traditional machine learning algorithms (logistic regression, support vector machine, random forest, and extreme gradient boosting) and one deep learning architecture (TabNet) were developed with Bayesian optimization for hyperparameter tuning. Model performance was assessed through comprehensive metrics including discrimination, calibration, clinical utility, and confusion matrices. The optimal model underwent feature importance analysis, systematic ablation studies, sensitivity analyses, and subgroup fairness evaluation.

**Results:**

Of 143,192 analyzed visits, 24,117 (16.8%) were classified as URVs. Data were allocated into training (*n* = 100,235) and testing (*n* = 42,957) sets with consistent URV proportions. TabNet demonstrated optimal discriminative performance with AUROC 0.867 (95% CI: 0.854–0.880) and sensitivity of 0.809 (95% CI: 0.801–0.816). Decision curve analysis demonstrated sustained clinical utility across threshold probabilities of 10–30%. Feature importance analysis identified initial diagnoses of digestive and respiratory system diseases, patient age, P3 triage classification, and ED visit frequency as key predictive variables. Subgroup analysis confirmed consistent performance across patient demographics and clinical characteristics.

**Conclusion:**

TabNet outperformed traditional machine learning approaches in predicting 72-h ED URVs, offering potential for improved risk stratification in emergency care settings.

## Introduction

Unscheduled return visits (URVs) to the emergency department (ED) within 72 h of initial discharge represent a critical healthcare quality metric. Global data demonstrate that 72-h URV rates vary from 0.4 to 15.8% across institutions (1), with studies indicating that 9 to 48% of these returns may be preventable through enhanced clinical processes (2). URVs carry significant clinical and financial implications, as returning patients may face increased health risks due to incomplete initial management, unrecognized diagnoses, or suboptimal discharge planning (3, 4). These potentially avoidable revisits contribute to unnecessary resource utilization, ED overcrowding, and increased healthcare costs (5, 6).

In response to these challenges, healthcare systems worldwide have prioritized URV monitoring and mitigation. The Centers for Medicare & Medicaid Services (CMS) in the United States has integrated ED revisit metrics into hospital performance assessment (7), while the Department of Health in the UK has incorporated URV rates into their quality framework to identify areas requiring intervention (8). These initiatives reflect the growing recognition that URV data analysis is fundamental to optimizing emergency care delivery. The ability to accurately predict high-risk patients enables targeted interventions for enhanced discharge safety and care coordination, ultimately leading to improved patient outcomes and reduced resource utilization (9, 10).

Numerous studies have explored factors contributing to ED URVs, categorizing them into four primary domains: patient demographics, clinical presentations, comorbidities, and system-related variables. Commonly identified predictors include advanced age, abdominal pain, and high triage acuity (11, 12). While these studies have enhanced our understanding of individual risk factors, their complex interactions and varying degrees of influence create significant challenges in accurately identifying high-risk patients. Traditional predictive models, primarily based on logistic regression (LR), have been widely utilized but exhibit limitations in handling nonlinear relationships and require strong assumptions about data distribution and independence (13). In recent years, artificial intelligence (AI)-based machine learning (ML) techniques have received increasing attention for their ability to identify intricate patterns within high-dimensional clinical data. Notable algorithms, including the support vector machine (SVM), random forest (RF), and extreme gradient boosting (XGBoost), have demonstrated moderate predictive performance for ED URVs, with a median area under the receiver operating characteristic curve (AUROC) of 0.73 and an interquartile range of 0.71–0.76 (14). Similar ML approaches have also gained recognition for their effectiveness in other ED applications, including length of stay prediction and admission forecasting, further establishing the potential of AI-driven solutions in emergency care settings (15). Beyond traditional ML approaches, recent advances in large language models (LLMs) have begun extending AI applications to emergency triage, demonstrating promising concordance with expert clinical judgment in triage classification (16–18).

Despite advancements in AI techniques, significant research gaps remain in the prediction of URVs. Most existing studies have employed a limited range of ML algorithms, often lacking comprehensive comparisons within the same cohort to determine relative model performance (14, 19, 20). Additionally, the potential of deep learning (DL) architectures, particularly TabNet which was specifically designed for tabular data analysis, remains unexplored in URV prediction. TabNet offers distinct computational characteristics in processing tabular data through its sequential self-attention mechanism that enables dynamic feature importance weighting at each decision step, theoretically different from traditional ML approaches that typically rely on predefined feature engineering and explicit model structures. However, whether these architectural differences translate to improved predictive performance in URV risk stratification requires systematic investigation.

To address these evidence gaps, this study aims to comprehensively evaluate and compare the predictive performance of multiple AI models for 72-h ED URV using a single-center cohort. We assessed five distinct algorithms: traditional ML approaches (LR, SVM, RF, and XGBoost) and TabNet. By elucidating optimal AI strategies for URV risk stratification, we seek to accelerate the deployment of robust, interpretable clinical decision support systems to enhance discharge planning and care transitions in emergency medicine practice.

## Materials and methods

This retrospective observational study was conducted in full compliance with the ethical principles outlined in the Declaration of Helsinki and adhered to the Transparent Reporting of a multivariable prediction model for Individual Prognosis or Diagnosis (TRIPOD) guidelines for prediction modeling studies. A comprehensive Prediction model Risk of Bias ASsessment Tool (PROBAST) evaluation was performed to assess methodological quality and potential sources of bias, with completed checklists provided in Supplementary materials S1, S2, respectively. Ethical approval was obtained from the Institutional Review Board of Shanghai East Hospital, School of Medicine, Tongji University (Approval No.: 2024YS-259). Due to the retrospective nature of the study, the requirement for informed consent was waived by the ethics committee. All data used in this analysis were anonymized to ensure participant confidentiality and privacy were rigorously protected. Comprehensive measures were implemented to prevent the identification of individual participants, maintaining adherence to ethical standards throughout the research process.

### Data source

This study utilized data from the ED electronic medical records of Shanghai East Hospital affiliated with Tongji University, a large tertiary teaching hospital with an advanced information management system. The dataset included all ED records from 1 January to 31 December 2023. Inclusion criteria comprised adult patients with medical ED visits. Exclusion criteria included pediatric cases, surgical cases, and index ED visits resulting in hospital admission, death, transfer to other facilities, or departure against medical advice. To minimize potential confounding from planned short-term revisits, patients with revisit intervals of 3 ≤ 𝑡_subsequent_ − 𝑡_initial_ ≤ 7 days were excluded. The remaining patients were categorized into two groups based on their revisit behavior following ED discharge. Those who did not return within 7 days were classified as the control group, while those who revisited the ED within 72 h of discharge (𝑡_subsequent_ − 𝑡_initial_ < 3 days) were classified as the URV group. For patients with multiple qualifying URV episodes during the study period, only the first occurrence was included. Variables extracted from the initial ED visit were grouped into four categories: demographic information, clinical characteristics, behavioral patterns, and hospital-specific details. All predictor variables were documented during the initial ED evaluation and were available before the discharge disposition decision. A comprehensive list of these variables, their definitions, and timing of availability is provided in Supplementary Table S1.

### Data splitting

The dataset was partitioned using stratified random sampling based on URV status to ensure proportional representation in both training (70%) and testing (30%) cohorts. This stratification procedure was implemented to maintain consistent URV distribution across the partitioned datasets. Standardized mean differences were calculated to assess the balance of baseline characteristics between cohorts, with values below 0.1 considered indicative of adequate distribution equilibrium.

### Data preprocessing

Data preprocessing followed a standardized four-step pipeline. First, missing data were handled using complete case analysis, excluding patients with missing values for any predictor variables. Second, variable encoding was performed: binary variables were processed through label encoding (0/1), ordinal variables (triage levels) were encoded using ordinal encoding to retain hierarchical structure, and the binary outcome was defined as 1 for URV group and 0 for control group. Third, continuous variables were standardized using z-score transformation. Fourth, class imbalance in the training dataset was addressed through a hybrid sampling approach combining ADASYN oversampling with TomekLinks undersampling to achieve balanced class distributions. All preprocessing steps were implemented using Python 3.11.4 with random seed 42 for reproducibility.

### Model development and hyperparameter optimization

To develop predictive models for URVs, four established supervised ML algorithms including LR, RF, SVM, and XGBoost, along with the DL model TabNet, were utilized. Model selection was guided by suitability for tabular clinical data and interpretability requirements for ED deployment. Traditional ML algorithms provide transparent decision pathways essential for clinical acceptance, while TabNet was selected as the DL representative due to its specific architecture for structured data processing through sequential attention mechanisms. The optimal hyperparameter settings were determined through Bayesian optimization coupled with 5-fold cross-validation, with 50 optimization trials conducted per model. During the optimization process, the hyperparameter configuration that achieved the minimum validation loss was selected as the optimal setting, as this criterion indicates the best model generalization capability and minimizes potential overfitting. In the TabNet implementation, training was terminated when no validation performance improvement could be detected across 10 consecutive epochs. After determining the best-performing hyperparameter configurations, the final models were trained on the complete training dataset using the optimized parameters.

### Model evaluation

Using the independent testing dataset, all models were evaluated through a comparative analysis to identify the one that demonstrated the most reliable performance in predicting URVs. Model performance was assessed based on discrimination, calibration, and clinical relevance. Standardized evaluation metrics were calculated based on confusion matrices. The AI model demonstrating superior overall performance across these multiple evaluation dimensions was selected as the optimal predictor and subjected to additional robustness testing. Four sensitivity analyses were conducted on the optimal model to evaluate performance stability under varied analytical conditions including alternative missing data handling through multiple imputation with chained equations, no-resampling training, temporal holdout validation using final quarter data, and analysis with imaging variables removed.

### Interpretation of the optimal model

The optimal performing model underwent systematic interpretability analysis through multiple validation approaches. Feature importance was quantified using model-specific attention mechanisms, with additional model-agnostic validation through permutation importance analysis across 1,000 bootstrap iterations to establish statistical robustness. Multicollinearity assessment among top predictors was performed using correlation analysis. Systematic ablation studies evaluated clinical significance by sequentially removing key predictive features and measuring performance degradation. Subgroup fairness assessment evaluated algorithmic equity across clinically relevant patient subgroups using coefficient of variation analysis to quantify performance consistency.

### Statistical analysis

Comparisons of characteristics between URV and control groups were performed using Mann–Whitney U test for continuous variables and chi-square test for categorical variables. For model evaluation, discrimination was assessed through ROC curve analysis, with the AUC used as the primary metric. Statistical comparisons between model AUCs were performed using DeLong’s test to assess significance of performance differences. Optimal decision thresholds were determined using Youden’s index to maximize overall classification performance, and confusion matrices were constructed at these thresholds to derive performance metrics including accuracy, precision, recall, F1-score and Kappa. Ninety-five percent confidence intervals (95% CIs) were calculated for all key performance metrics to quantify uncertainty. Calibration performance was assessed through calibration curves and quantitative metrics including Brier scores, and calibration intercept, and slope derived from logistic regression of observed outcomes against predicted probabilities. Expected Calibration Error (ECE) was computed as the weighted average of absolute calibration errors across probability bins to provide comprehensive calibration assessment. To determine the clinical relevance of the models, decision curve analysis (DCA) was performed to calculate net benefit across various threshold probabilities.

## Results

### Study population characteristics

**Figure 1:**
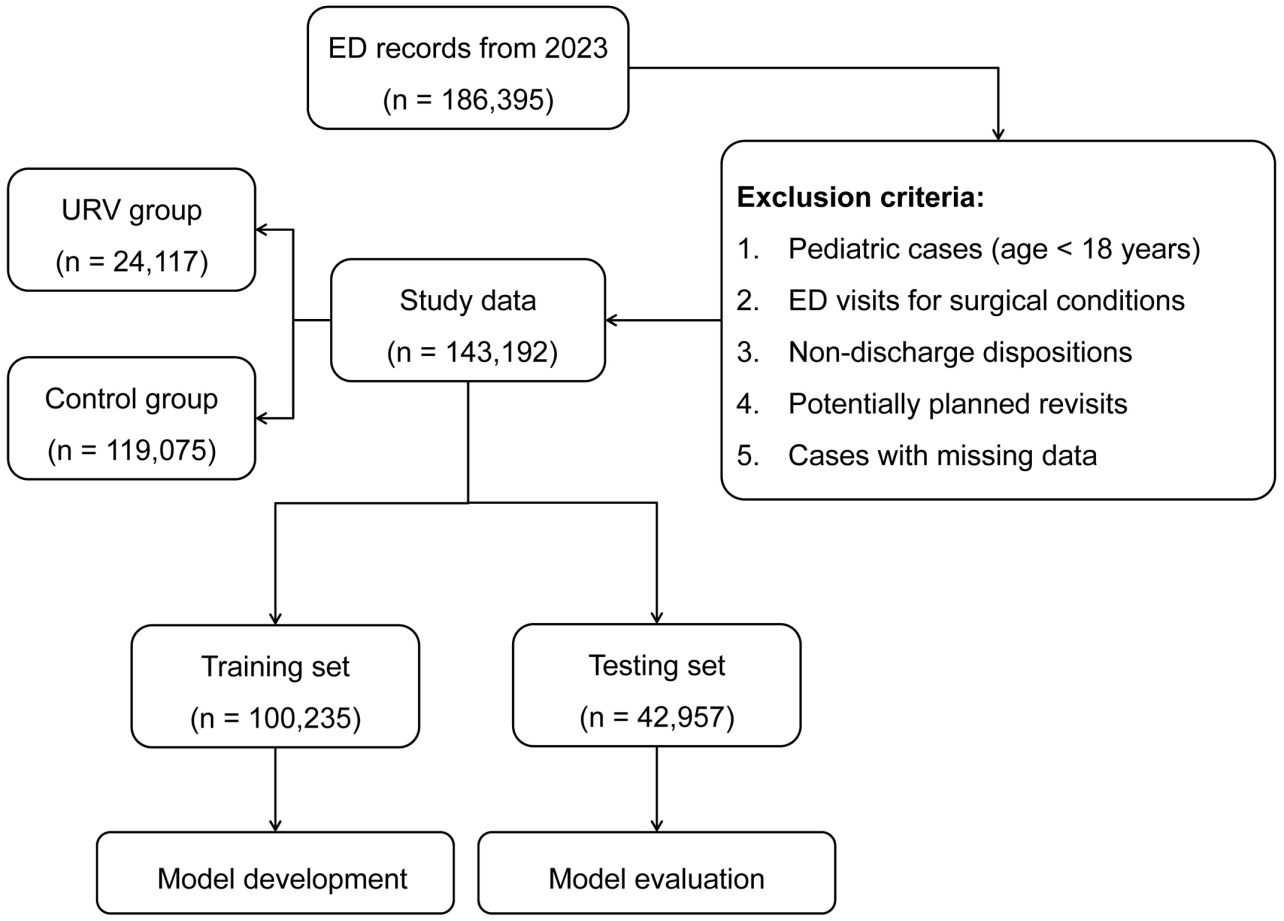
Flowchart of data stratification and study design.

Figure 1 presents a comprehensive flowchart depicting the data selection process and analytical framework of our investigation. After data cleaning and preprocessing, the final dataset consisted of 143,192 patients, with 24,117 (16.8%) allocated to the URV group and 119,075 (83.2%) to the control group. The initial dataset contained 186,395 ED records. After applying inclusion and exclusion criteria and removing cases with missing data (overall missing rate 6.8%), the final dataset consisted of 143,192 patients, with 24,117 (16.8%) allocated to the URV group and 119,075 (83.2%) to the control group. Variable-specific missing rates were below 1.5% for most variables, with missing data primarily concentrated in blood pressure measurements (8.7%) and initial diagnosis categories (4.3%). Table 1 presents the characteristics of the URV and control groups at the initial ED visit. Due to the large sample sizes in both groups, all examined characteristics exhibited statistically significant differences between the URV and control groups (all *p* < 0.05). The dataset was further divided into a training set of 100,235 patients and a testing set of 42,957 patients. The proportion of URV cases remained consistent between the training (16.8%, 16,882 patients) and testing sets (16.8%, 7,235 patients), ensuring balanced distribution for model development and evaluation.

**Table 1:** Baseline characteristics of the URV and control groups at the initial ED visit.

Characteristic		Control group (*n* = 119,075)	URV group (*n* = 24,117)	*P* value
Age, years		45 (33, 67)	66 (42, 76)	<0.001
Gender, *n* (%)	Male	54,110 (45.4%)	10,766 (44.6%)	0.023
	Female	64,965 (54.6%)	13,351 (55.4%)	
Common medical history, *n* (%)	Hypertension	20,389 (17.1%)	6,873 (28.4%)	<0.001
	Diabetes	16,564 (13.9%)	5,940 (24.6%)	<0.001
	Cardiovascular disease	7,652 (6.4%)	2,894 (11.9%)	<0.001
	Hyperlipidemia or fatty liver	8,611 (7.2%)	3,656 (15.1%)	<0.001
	Stroke	1,955 (1.6%)	635 (2.6%)	<0.001
Body temperature, °C		37.2 (36.9, 37.5)	37.7 (36.9, 38.2)	<0.001
Systolic blood pressure, mmHg		122.9 (116.2, 129.7)	128.3 (121.3, 134.2)	<0.001
Diastolic blood pressure, mmHg		81.9 (75.2, 88.7)	82.4 (75.3, 89.3)	0.014
Heart rate, beats/min		79 (73, 86)	81 (73, 90)	0.011
Blood oxygen saturation, %		98 (96, 99)	98 (95, 99)	0.015
Activity ability, *n* (%)	Independent	80,520 (67.6%)	12,132 (50.3%)	
	Accompanied	28,435 (23.8%)	9,060 (37.6%)	
ED visits last month, *n*		1 (0, 1)	1 (0, 2)	<0.001
Initial triage level, *n* (%)	P1	13,824 (11.6%)	1,063 (4.4%)	<0.001
	P2	18,218 (15.3%)	2,170 (9.0%)	
	P3	33,329 (28.0%)	12,741 (52.8%)	
	P4	41,473 (34.8%)	5,686 (23.6%)	
Diagnostic procedures, *n* (%)	CT	65,564 (55.0%)	20,003 (82.9%)	<0.001
	Ultrasound	34,867 (29.2%)	11,406 (47.2%)	<0.001
	ECG	48,912 (41.0%)	15,308 (63.4%)	<0.001
	MRI	18,898 (15.8%)	6,414 (26.5%)	<0.001
Initial diagnosis, *n* (%)	Respiratory system disease	33,036 (27.7%)	8,864 (36.8%)	<0.001
	Cardiovascular system disease	6,302 (5.2%)	965 (4.0%)	
	Digestive system disease	35,453 (29.8%)	8,948 (37.1%)	
	Nervous system disease	18,053 (15.2%)	2,160 (9.0%)	
	Urinary system disease	14,332 (12.0%)	2,200 (9.1%)	
Physician shift, *n* (%)	Morning	50,391 (42.3%)	11,614 (48.1%)	<0.001
	Afternoon	42,371 (35.5%)	7,246 (30.0%)	
	Night	26,313 (22.0%)	5,257 (21.7%)	
Holiday status at initial visit, *n* (%)		35,834 (30.0%)	6,100 (25.3%)	<0.001
Physician seniority, *n* (%)	Attending Physician	63,593 (53.4%)	15,154 (62.8%)	<0.001
	Associate Senior Physician	19,379 (16.2%)	3,948 (16.3%)	
	Senior Physician	2,544 (2.1%)	575 (2.3%)	

Missing data were not included in the presented analyses.

### Data preprocessing

To address the class imbalance inherent in the training dataset, we implemented a hybrid sampling approach combining ADASYN oversampling with TomekLinks undersampling techniques. This data balancing strategy yielded a final training set of 148,692 cases, with equal distribution between URV and control groups (74,346 cases each), facilitating optimal feature learning from the minority class.

### Hyperparameter optimization and model development

Five models were developed to predict URV risk, including four ML algorithms (LR, SVM, RF, and XGBoost) and one DL architecture (TabNet). The optimal hyperparameter configurations for each model were identified through Bayesian optimization with 50 trials, selecting configurations that achieved minimum validation loss as the optimization objective (Supplementary Figure S1). The detailed hyperparameter settings that achieved optimal performance can be found in Supplementary Table S2. These optimized configurations were then applied to train the final models.

### Model performance evaluation

**Figure 2:**
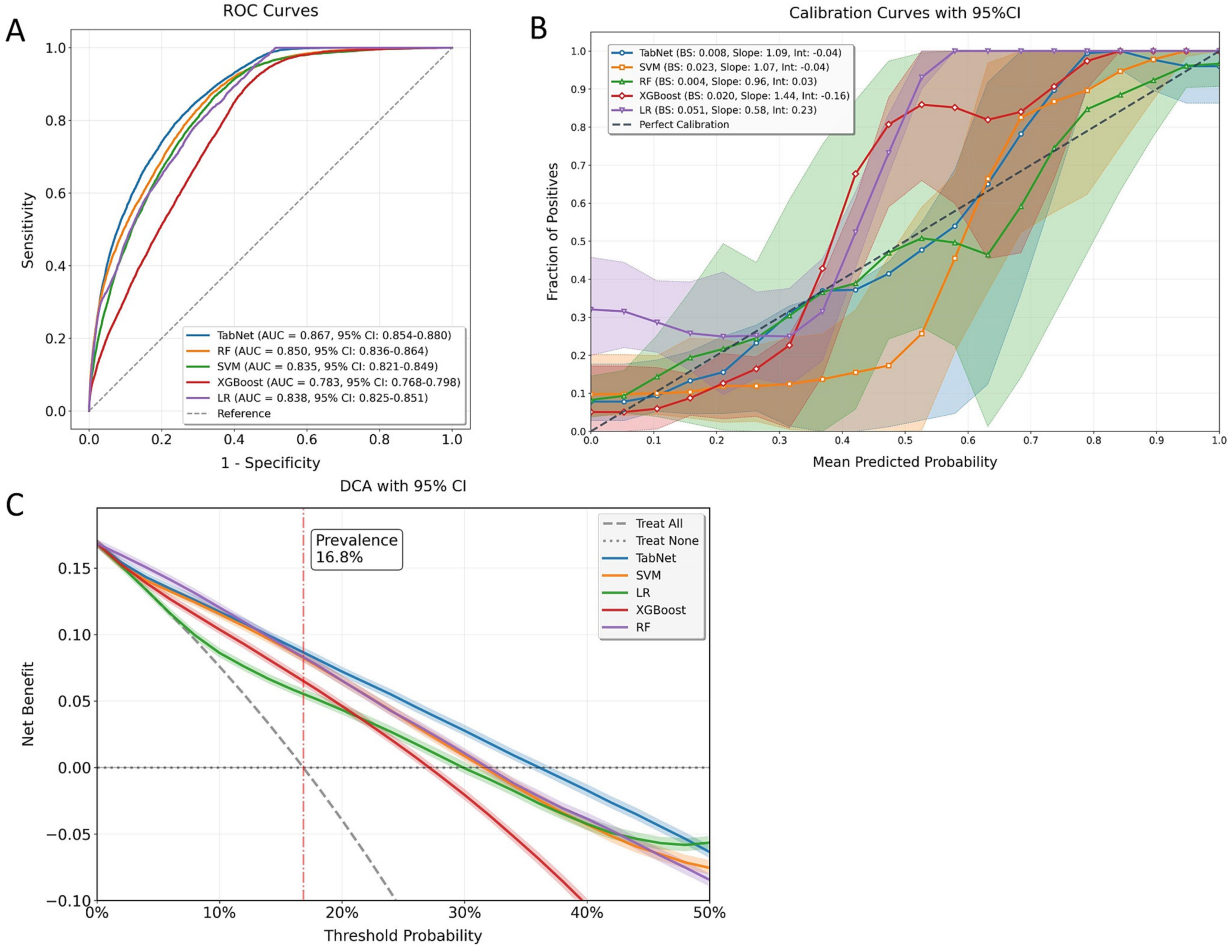
Comprehensive model evaluation metrics. **(A)** ROC curves displaying discrimination ability of different models. **(B)** Calibration plots showing the relationship between predicted and observed probabilities with corresponding Brier scores. **(C)** DCA demonstrating clinical utility across different threshold probabilities.

The predictive capabilities of all models were evaluated on an independent testing set to validate their performance in URV identification. Comprehensive model assessment was conducted through discrimination, calibration, and clinical utility metrics visualized in Figure 2. TabNet and RF demonstrated superior discriminative performance, with both achieving AUROC values above 0.85, significantly higher than the other three ML models (all *p* < 0.05, DeLong’s test). Calibration performance was assessed through calibration curves and quantitative calibration metrics including Brier scores, intercept, and slope parameters. ECE was computed as the weighted average of absolute calibration errors across probability bins to provide comprehensive calibration assessment (Supplementary Table S3). RF demonstrated the most favorable calibration profile with minimal systematic bias and the lowest ECE, while TabNet showed acceptable calibration performance with slightly higher deviation in the mid-probability ranges. Both models outperformed approaches, with SVM, XGBoost, and LR exhibiting greater calibration errors. DCA across clinically relevant threshold probabilities (10–30%) demonstrated varying model performance patterns (Supplementary Table S4). TabNet exhibited superior and consistent clinical utility throughout the evaluated threshold range. While competing models showed performance degradation at higher thresholds, TabNet maintained sustained net benefit across the spectrum of clinically relevant decision points.

**Figure 3:**
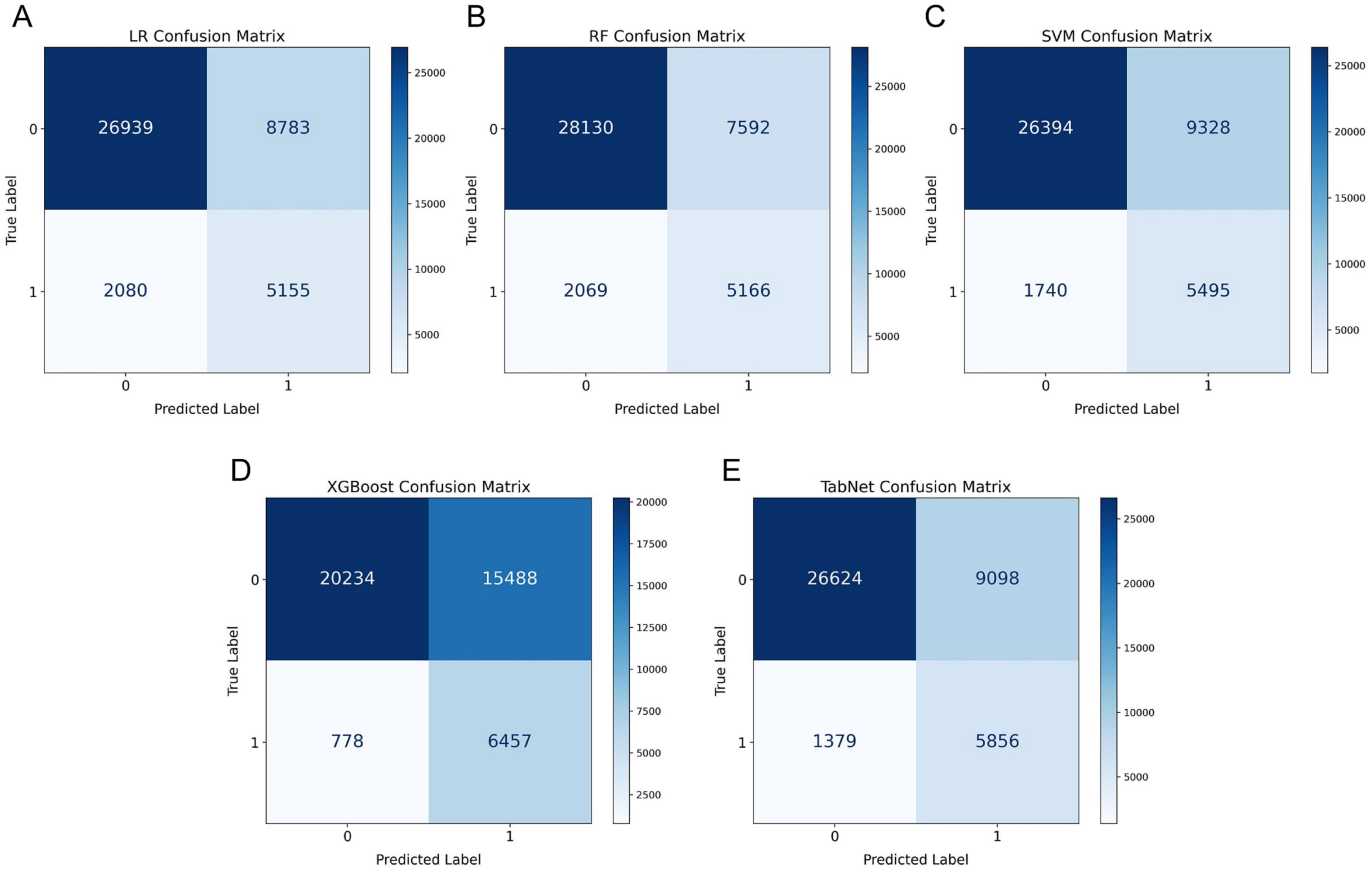
Confusion matrices for model performance comparison. **(A)** LR, **(B)** RF, **(C)** SVM, **(D)** XGBoost, and **(E)** TabNet. The matrices display true negatives (top left), false positives (top right), false negatives (bottom left), and true positives (bottom right).

Further analysis through confusion matrices (Figure 3) and performance metrics (Table 2) confirmed the superior performance of TabNet and RF algorithms. Notably, TabNet demonstrated enhanced sensitivity in URV detection with a recall rate of 0.809, failing to identify only 1,379 cases compared to 2,069 cases missed by RF. These results establish TabNet as the optimal predictive model for URV risk stratification.

**Table 2:** Performance metrics of different models in the testing set.

Model	Optimal threshold	TP	FP	TN	FN	Accuracy	Precision	Recall	F1 score	Kappa
LR	0.52	5,155	8,783	26,939	2,080	0.747 (0.741, 0.753)	0.370 (0.359, 0.381)	0.713 (0.701, 0.725)	0.487 (0.477, 0.497)	0.341 (0.331, 0.351)
RF	0.48	5,166	7,592	28,130	2,069	0.775 (0.770, 0.779)	0.405 (0.397, 0.413)	0.714 (0.705, 0.722)	0.517 (0.510, 0.524)	0.384 (0.377, 0.391)
SVM	0.50	5,495	9,328	26,394	788	0.742 (0.736, 0.748)	0.371 (0.361, 0.382)	0.760 (0.749, 0.771)	0.498 (0.488, 0.507)	0.350 (0.340, 0.360)
XGBoost	0.42	6,457	15,488	20,234	778	0.621 (0.614, 0.628)	0.294 (0.283, 0.306)	0.892 (0.885, 0.898)	0.443 (0.434, 0.453)	0.253 (0.242, 0.264)
TabNet	0.46	5,856	9,098	26,624	1,379	0.756 (0.751, 0.760)	0.392 (0.385, 0.400)	0.809 (0.801, 0.816)	0.528 (0.522, 0.535)	0.388 (0.382, 0.395)

TP, true positive; FP, false positive; TN, true negative; FN, false negative. Values in parentheses represent 95% CI.

### Model robustness evaluation

Four sensitivity analyses evaluated TabNet robustness under varied analytical constraints (Table 3). Multiple imputation analysis incorporating the 6.8% cases with missing data yielded performance metrics comparable to the original complete case approach, with overlapping confidence intervals confirming that missing data handling did not substantially affect model conclusions. No-resampling training demonstrated substantial performance degradation with severe recall decline, confirming the necessity of balanced training for minority class detection in imbalanced medical datasets. Temporal holdout validation using the final quarter (October–December 2023) revealed minimal performance decline despite three-month separation, indicating robust discriminative capability across patient population drift and seasonal variations. Removing imaging variables produced minimal impact, demonstrating preserved predictive performance when restricted to early triage features.

**Table 3:** TabNet performance under sensitivity analysis conditions.

Condition	Accuracy	Precision	Recall	F1-score
Original	0.756 (0.751, 0.760)	0.392 (0.385, 0.400)	0.809 (0.801, 0.816)	0.528 (0.522, 0.535)
Multiple imputation	0.752 (0.747, 0.757)	0.388 (0.381, 0.396)	0.806 (0.798, 0.814)	0.524 (0.518, 0.531)
No-resampling training	0.697 (0.691, 0.703)	0.407 (0.396, 0.419)	0.649 (0.637, 0.661)	0.509 (0.500, 0.518)
Temporal holdout	0.738 (0.731, 0.745)	0.391 (0.381, 0.402)	0.777 (0.766, 0.788)	0.522 (0.513, 0.531)
No imaging variables	0.748 (0.742, 0.754)	0.391 (0.383, 0.399)	0.795 (0.786, 0.803)	0.528 (0.521, 0.536)

Values in parentheses represent 95% CI.

### Interpretation of the optimal model

**Figure 4:**
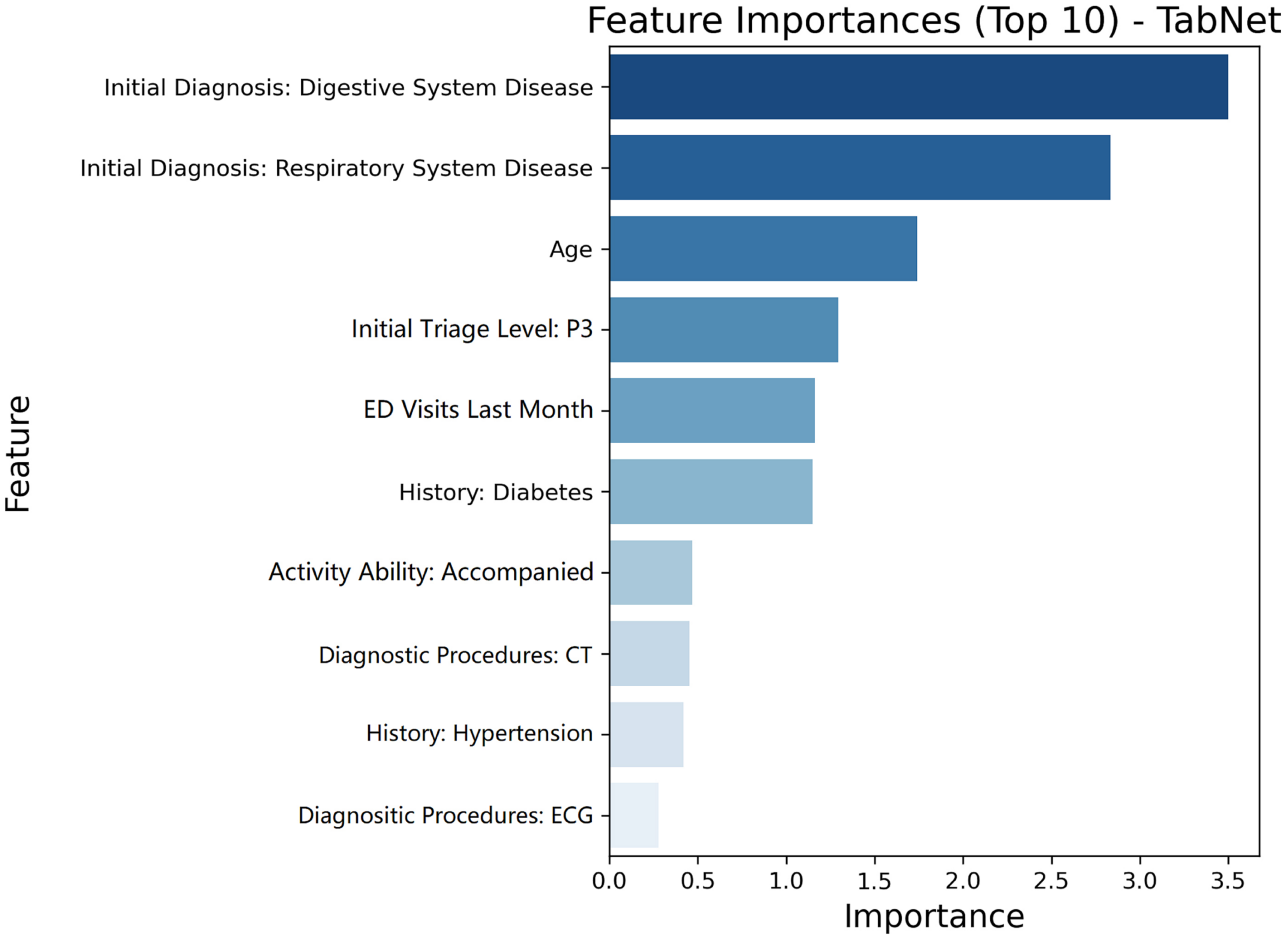
Feature importance ranking derived from TabNet attention weights showing the top 10 predictive variables for 72-h ED URV. Importance scores are normalized attention weights ranging from 0 to 1, with higher values indicating greater contribution to model predictions.

**Figure 5:**
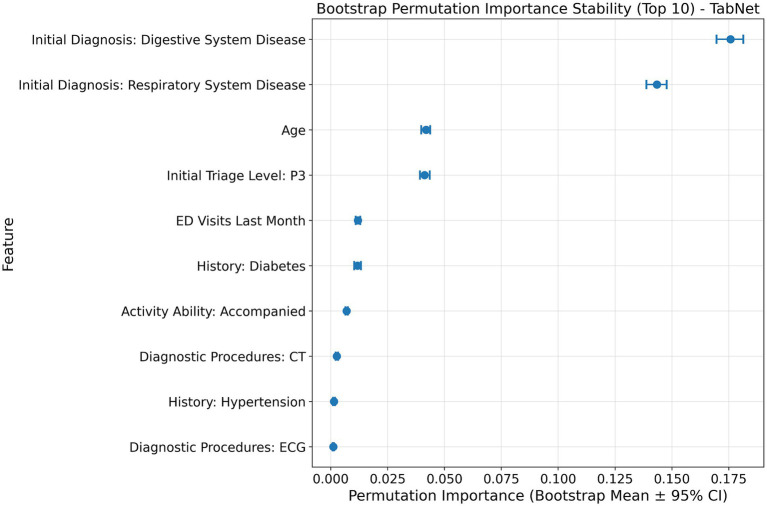
Bootstrap permutation importance stability analysis for TabNet URV prediction. Permutation importance values with 95% confidence intervals across 1,000 bootstrap iterations for the top 10 predictive features, demonstrating statistical robustness of feature rankings.

**Figure 6:**
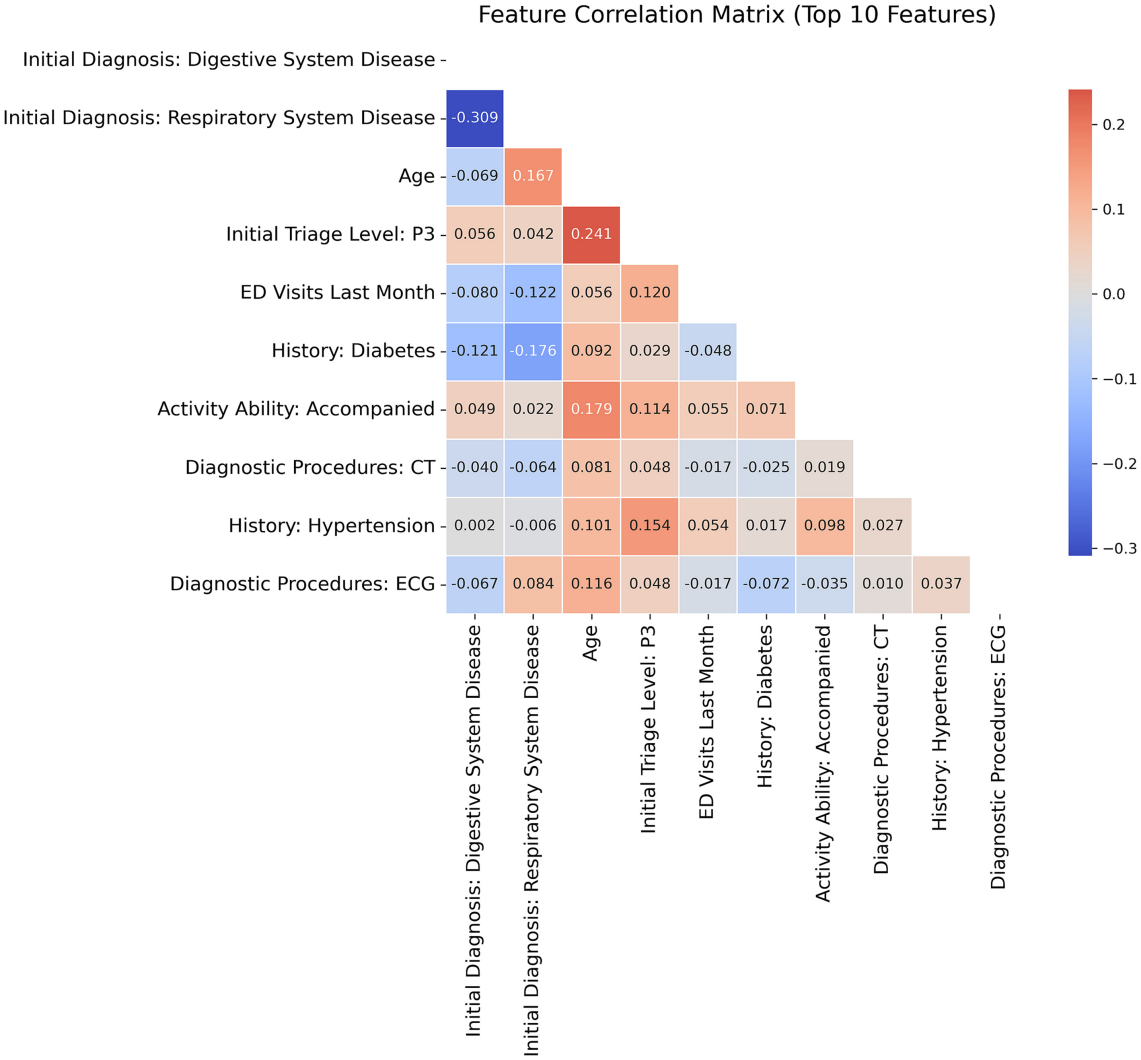
Correlation matrix of top 10 predictive features. Heat map displaying pairwise Pearson correlation coefficients ranging from −1 to +1, with color intensity indicating correlation strength. All correlations below 0.3 threshold confirm absence of multicollinearity.

Feature importance analysis of TabNet revealed significant predictive patterns for URV risk stratification through multiple validation approaches. Variable contribution assessment identified ten key predictors quantified through attention mechanisms (Figure 4). Model-agnostic validation using permutation importance with bootstrap stability analysis across 1,000 iterations established statistical robustness, showing consistent importance hierarchies with narrow confidence intervals (Figure 5). Digestive and respiratory system diagnoses demonstrated the highest predictive contributions in both attention-based and permutation-based assessments. Correlation analysis among top predictors revealed no significant multicollinearity (Figure 6), with all pairwise correlations remaining below 0.3, effectively ruling out redundant or artificially inflated feature contributions.

Systematic ablation analysis validated the clinical significance of identified predictors through progressive performance deterioration (Table 4). Sequential elimination of digestive system diagnosis caused the most substantial impact, reducing model accuracy from 0.756 to 0.643 and F1 score from 0.528 to 0.391. Subsequent removal of respiratory system diagnosis, age, P3 triage classification, and ED visit frequency produced incrementally smaller performance decrements, primarily attributed to compromised sensitivity as evidenced by declining recall values.

**Table 4:** Sequential ablation analysis demonstrating the impact of key predictor removal on TabNet model performance metrics.

Model	Accuracy	Precision	Recall	F1 Score	Kappa
Original	0.756 (0.751, 0.760)	0.392 (0.385, 0.400)	0.809 (0.801, 0.816)	0.528 (0.522, 0.535)	0.388 (0.382, 0.395)
Without initial diagnosis: digestive system disease	0.643 (0.635, 0.651)	0.312 (0.301, 0.324)	0.524 (0.511, 0.538)	0.391 (0.380, 0.403)	0.090 (0.078, 0.102)
Without initial diagnosis: respiratory system disease	0.681 (0.674, 0.688)	0.339 (0.329, 0.350)	0.576 (0.564, 0.588)	0.432 (0.422, 0.442)	0.146 (0.136, 0.157)
Without age	0.706 (0.700, 0.712)	0.358 (0.349, 0.367)	0.665 (0.654, 0.676)	0.465 (0.456, 0.474)	0.213 (0.204, 0.222)
Without initial triage level: P3	0.724 (0.719, 0.729)	0.371 (0.363, 0.379)	0.717 (0.707, 0.727)	0.489 (0.481, 0.497)	0.261 (0.253, 0.269)
Without ED visits last month	0.741 (0.736, 0.746)	0.381 (0.373, 0.389)	0.766 (0.757, 0.775)	0.509 (0.501, 0.517)	0.307 (0.299, 0.315)

Values in parentheses represent 95% CI.

### Subgroup fairness assessment

Based on the identified key predictive variables, subgroup analysis evaluated algorithmic fairness across age, triage level, and initial diagnosis categories (Table 5). TabNet maintained consistent recall performance (0.799–0.830) across all subgroups, ensuring equitable high-risk patient identification. Age-stratified analysis revealed accuracy declining from 0.839 in younger adults to 0.664 in older patients, a 17.5 percentage point differential within clinically acceptable limits. Performance across diagnostic categories (accuracy 0.744–0.896) and triage levels demonstrated variations consistent with clinical complexity expectations. Coefficient of variation analysis confirmed good fairness for diagnostic categories (7.8%) and acceptable fairness for age groups and triage levels (10.4 and 11.2% respectively), supporting equitable clinical deployment.

**Table 5:** Subgroup performance analysis of TabNet model across patient demographics and clinical characteristics.

Analysis type	Subgroup	Sample size (*n*)	URV (*n*)	URV rate (%)	Accuracy	Precision	Recall	F1 score
Original		42,958	7,235	16.8	0.756 (0.751, 0.761)	0.392 (0.385, 0.400)	0.809 (0.801, 0.816)	0.528 (0.522, 0.535)
Age groups	18–40 years	13,301	860	6.5	0.839 (0.832, 0.846)	0.263 (0.247, 0.280)	0.828 (0.803, 0.851)	0.398 (0.382, 0.415)
	41–65 years	12,100	1,566	12.9	0.799 (0.791, 0.807)	0.373 (0.354, 0.393)	0.814 (0.794, 0.833)	0.511 (0.494, 0.528)
	>65 years	17,556	4,809	27.4	0.664 (0.657, 0.671)	0.438 (0.425, 0.451)	0.805 (0.793, 0.817)	0.568 (0.557, 0.579)
Triage levels	P1	4,453	5	0.1	0.980 (0.976, 0.983)	0.043 (0.014, 0.104)	0.800 (0.374, 0.968)	0.082 (0.027, 0.186)
	P2	7,326	746	10.2	0.818 (0.808, 0.827)	0.336 (0.309, 0.365)	0.802 (0.772, 0.829)	0.473 (0.449, 0.498)
	P3	14,063	4,112	29.2	0.758 (0.751, 0.765)	0.479 (0.466, 0.492)	0.799 (0.786, 0.811)	0.599 (0.588, 0.610)
	P4	17,115	2,372	14.4	0.845 (0.839, 0.851)	0.466 (0.449, 0.484)	0.830 (0.813, 0.846)	0.597 (0.583, 0.611)
Initial diagnosis	Respiratory system disease	19,779	3,579	18.1	0.746 (0.740, 0.753)	0.399 (0.384, 0.414)	0.803 (0.790, 0.816)	0.533 (0.521, 0.545)
	Cardiovascular system disease	5,262	413	7.8	0.885 (0.875, 0.894)	0.387 (0.349, 0.427)	0.804 (0.764, 0.840)	0.523 (0.490, 0.556)
	Digestive system disease	12,221	2,839	23.2	0.744 (0.736, 0.752)	0.468 (0.451, 0.485)	0.800 (0.783, 0.816)	0.590 (0.576, 0.604)
	Nervous system disease	1,187	103	8.7	0.857 (0.834, 0.877)	0.359 (0.293, 0.431)	0.825 (0.740, 0.889)	0.500 (0.437, 0.563)
	Urinary system disease	4,508	301	6.7	0.896 (0.886, 0.905)	0.373 (0.325, 0.424)	0.817 (0.770, 0.857)	0.513 (0.472, 0.554)

Values in parentheses represent 95% CI.

## Discussion

Current prediction models for ED URVs demonstrate limited performance, primarily due to insufficient understanding of the specific strengths and applications of different AI architectures in clinical data analysis. Our study addressed this limitation by conducting the first comprehensive comparison of multiple AI architectures within a large unified cohort of ED visits, enabling direct performance assessment under identical conditions. The results demonstrated that TabNet, a DL framework designed for tabular data analysis through self-attention mechanisms and adaptive feature selection, achieved superior predictive performance (AUROC 0.85, recall 0.809) compared to ML models with fixed architectural frameworks. This comparative analysis revealed substantial performance variations among AI approaches, with TabNet’s dynamic feature-learning capabilities proving particularly well-suited for URV prediction. These findings not only establish TabNet as an optimal choice for ED URV risk stratification but also provide valuable insights into the specific advantages of different AI architectures in clinical prediction tasks, offering a promising direction for enhancing URV risk identification in emergency care settings.

### Clinical significance and healthcare burden

The accurate prediction of URV risk carries significant implications for healthcare delivery optimization and patient safety enhancement. Our observed URV rate of 16.8% substantially exceeds rates reported from comparable tertiary institutions in regions with established tiered healthcare systems, including Singapore (1.9–2.9%) (5, 11), Canada (8.7%) (3), and the United States (3.6–8.8%) (6, 10). This elevated proportion underscores the critical importance of URV prediction in the Chinese healthcare context, as the developing tiered care system and direct patient preference for tertiary hospitals create a substantial and persistent burden that cannot be rapidly restructured through policy interventions alone.

The 72-h temporal threshold represents the established international standard for URV monitoring, providing optimal balance between capturing clinically significant returns while maintaining administrative feasibility across healthcare systems (21). URVs represent a significant patient safety concern, as studies demonstrate that patients returning within 72 h face substantially elevated risks compared to initial visits, with high-risk URVs requiring hospital admission showing mortality rates of 0.7% and intensive care unit admission rates of 11.1% (22). Among URV patients subsequently requiring ICU care, diagnostic error rates from the index visit may exceed 40%, highlighting the clinical significance of accurate initial assessment and disposition decisions (23). Each URV essentially doubles ED resource utilization (24), with the average visit requiring approximately 4 h of clinical care according to Di Somma et al. (25).

Our finding that P3 patients demonstrated the highest URV rates aligns with international observations that intermediate-acuity patients occupy a clinical vulnerability zone where symptom complexity may be systematically underestimated during initial evaluation, despite subsequently manifesting serious conditions requiring intensive intervention upon return (26). This contrasts with high-acuity patients who receive comprehensive monitoring and low-acuity cases requiring minimal intervention. If these patients could be identified and receive enhanced care planning, including comprehensive discharge instructions, medication counseling, and appropriate follow-up arrangements, many unnecessary returns could be prevented. From a patient-centric perspective, accurate URV prediction enables proactive care planning, potentially reducing adverse outcomes associated with delayed treatment (27, 28). Enhanced discharge planning for high-risk patients may improve treatment adherence, reduce anxiety about symptom recurrence, and strengthen patient trust in healthcare providers through improved discharge safety protocols (29, 30).

### Comparative performance analysis of AI architectures

Five distinct AI models were evaluated in this study, encompassing both potential linear and nonlinear relationships in URV prediction. Comparative analysis revealed marked variations in model performance. XGBoost exhibited the lowest discriminative ability with an AUROC of 0.621 and poor precision (0.294), indicating considerable false-positive predictions despite high sensitivity. This suboptimal performance may be attributed to XGBoost’s sequential tree-building process, which can be sensitive to noise in clinical data and prone to overfitting when handling complex temporal patterns characteristic of ED visits (31). LR and SVM demonstrated similar discriminative capabilities (AUROC 0.838 and 0.835, respectively), but LR was characterized by poor calibration. This limitation was attributed to the inherent linear relationship assumptions in LR, which were inadequate for capturing intricate patterns in URV risk factors (13). RF and TabNet were identified as superior predictive frameworks, both achieving AUROC values above 0.85. The robust performance was attributed to their architectural advantages in processing tabular clinical data. RF demonstrated effectiveness through ensemble decision trees that capture complex variable interactions, while TabNet utilized self-attention mechanisms for adaptive feature processing (32, 33). Although both models demonstrated strong performance, TabNet exhibited higher recall (0.809 versus 0.714) compared to RF, indicating enhanced capability in identifying potential URV cases. This improved sensitivity was particularly valuable for URV prediction, where identifying high-risk patients for preventive intervention remains a primary clinical objective.

These findings establish TabNet as the best-performing predictive model for URV risk stratification in our comparative analysis. TabNet represents a DL architecture specifically designed for tabular data that employs sequential attention mechanisms and learnable feature selection masks to automatically identify the most relevant predictive variables at each decision step (33). This architectural specialization enables superior performance in processing structured clinical data including demographics, vital signs, and diagnostic codes that are natively stored in electronic medical record systems. Unlike traditional neural networks that process all features simultaneously, TabNet’s multi-step attention process provides adaptive feature importance weighting while preserving interpretable attention weights, making it particularly well-suited for clinical applications where both predictive accuracy and model transparency remain essential for ED implementation. In contrast, LLMs are primarily optimized for natural language processing tasks and excel in scenarios requiring semantic understanding of unstructured text (16). Additionally, the institutional isolation of medical data due to privacy regulations and data governance policies constrains the availability of large-scale multi-institutional datasets that language model training typically requires, presenting practical barriers to LLM deployment in URV prediction contexts where structured tabular data predominates.

### Literature review

Recent investigations of ML-based URV prediction have predominantly utilized traditional algorithms with moderate performance outcomes. A comprehensive 2024 scoping review by Lee et al. (14) analyzed 14 recent implementations and confirmed that most studies remained constrained to conventional ML approaches using structured clinical variables from single institutions. The majority of these investigations focused on 72-h prediction windows using LR, XGBoost, and ensemble voting classifiers, achieving median AUROCs ranging from 0.66 to 0.74 (6, 14, 20, 34). These studies consistently demonstrated a fundamental challenge in URV prediction, specifically achieving optimal balance between sensitivity and specificity. Representative of this limitation, Hsu et al. (20) employed ensemble voting classifiers specifically for abdominal pain patients, achieving AUROC 0.74 with remarkably high specificity (0.89) but severely limited sensitivity (0.39). This pattern of sensitivity-specificity trade-offs appears across URV prediction studies, where traditional ML models either miss substantial proportions of true URV cases or generate excessive false positives that compromise clinical utility (6, 12, 20). To address these methodological limitations, our TabNet implementation utilizing a large-scale dataset of 143,192 patients achieved AUROC 0.867 with sensitivity of 80.9% and specificity of 74.5%, representing a substantial improvement over the reported literature median. This performance enhancement can be attributed to TabNet’s sequential attention mechanisms that enable dynamic feature importance weighting, contrasting with the fixed architectural frameworks that constrain traditional ML models. Notably, this investigation presents the first systematic application of DL architecture specifically designed for tabular data in URV prediction, addressing a critical methodological gap identified in the current literature.

### Feature importance and clinical risk factors

Feature importance analysis of the optimal model revealed several critical predictors for URV risk. Advanced age was identified as a predominant factor, which aligns with multiple previous studies that have documented increased URV risk in older patients, attributed to complex comorbidities, atypical disease presentations, and communication challenges (35–37). Initial diagnoses of respiratory and digestive system diseases were identified as significant risk factors, primarily due to non-specific presenting symptoms and condition complexity. Respiratory conditions often present with symptoms such as cough, dyspnea, or chest discomfort that lack diagnostic specificity and may represent diverse pathologies ranging from benign viral infections to severe pneumonia or pulmonary embolism. Similarly, digestive system diseases frequently manifest through non-specific complaints particularly abdominal pain, which encompasses a broad spectrum of etiologies from self-limited gastroenteritis to acute surgical emergencies, with symptom severity and clinical findings often insufficient to definitively establish diagnosis during initial ED evaluation. These conditions present diagnostic challenges through symptom overlap, multifaceted pathologies, and communication barriers between patients and healthcare providers (38).

The identification of P3 triage classification as a significant predictor differed from previous reports and warrants particular attention (39). This observation indicates that while P1 and P2 patients receive comprehensive evaluation due to the critical nature of their conditions, P3 patients, who constitute a substantial proportion of ED visits, may receive less intensive monitoring despite their complex medical presentations. In the high-volume ED environment, these intermediate-acuity patients might not receive the same level of detailed symptom assessment and continuous observation as higher-acuity cases, potentially leading to missed clinical deterioration or incomplete diagnosis. This finding emphasizes the importance of developing targeted assessment protocols for P3 patients during triage to better identify those at elevated URV risk, particularly given the diverse pathology and symptom complexity often present in this triage category. If high-risk P3 patients identified through predictive models could receive enhanced discharge planning including comprehensive instructions, medication counseling, and appropriate follow-up arrangements, many unnecessary returns could potentially be prevented.

Finally, ED visit frequency in the previous month was identified as a crucial predictor, indicating patient-specific factors including health anxiety, healthcare system navigation difficulties, and treatment plan adherence issues (40). These behavioral patterns highlight the necessity for targeted patient education and support systems to enhance health literacy and care plan adherence.

### Study limitations

This study presents notable methodological advances in URV prediction through comprehensive AI model comparison and the novel application of TabNet architecture in emergency medicine. Nevertheless, several limitations should be addressed in future investigations.

First, the single-center retrospective design with an observed URV rate of 16.8% exceeding international benchmarks from tertiary institutions limits generalizability to settings with different patient care-seeking behaviors and healthcare system structures. Additional methodological constraints include potential URV cases at other facilities, URV identification through administrative records rather than direct patient inquiry about the unscheduled nature of their return visits, inability to capture critical psychological factors and physician-patient communication quality that influence return decisions, absence of free-text clinical narratives such as patient chief complaints and physician documentation that might provide additional predictive information, absence of standardized discharge disposition documentation including follow-up recommendations in our electronic medical record system, and restriction to adult internal medicine cases which limits applicability to pediatric and surgical populations with distinct clinical characteristics (41). While the 72-h temporal criterion represents the established international standard, the gold standard for URV identification requires prospective validation through structured patient interviews at the time of return visits to confirm the unscheduled nature of their presentations.

Second, focusing on only the first URV episode per patient may not capture recurrent patterns that could enhance predictive modeling accuracy. The hybrid ADASYN-TomekLinks resampling strategy, while enhancing model sensitivity for minority class detection, generates synthetic samples that may not perfectly reflect true clinical patterns, though our sensitivity analysis without resampling confirmed the necessity of balanced training for effective URV identification. Furthermore, the P1 triage subgroup in our fairness assessment contained only 5 URV cases among 4,453 visits, yielding wide confidence intervals that limit reliable performance estimation for this highest-acuity category.

Third, practical implementation of TabNet in real-world ED settings presents technical and operational challenges that require systematic consideration. The model necessitates adequate computational infrastructure for real-time inference with latency requirements below 5 s to support triage workflow, alongside ongoing maintenance protocols to address potential data drift over time. Integration with existing hospital information systems demands careful attention to workflow compatibility, data security requirements, and compliance with healthcare data protection regulations. Staff training for clinical interpretation of model outputs, change management strategies to facilitate adoption, and technical support infrastructure represent additional barriers to implementation, particularly in resource-constrained healthcare environments. Future prospective deployment studies should systematically evaluate these operational requirements and establish evidence-based implementation frameworks.

### Future implementation strategies

Future research directions should focus on three key areas that leverage the clinical advantages identified through our TabNet implementation. First, prospective multicenter studies incorporating real-time patient interviews to validate URV classification accuracy and explore unmeasured behavioral determinants across diverse patient populations are essential. These studies should implement the model within triage protocols to identify high-risk patients based on presenting symptoms, age, triage classification, and recent ED utilization patterns, enabling automated risk assessment that operates alongside standard patient evaluation procedures by processing readily available demographic data, presenting complaints, and vital signs to provide immediate stratification alerts that support discharge planning decisions without disrupting established clinical workflows.

Second, external validation studies across diverse healthcare settings are needed to confirm model robustness and generalizability beyond the current institutional context, with particular attention to how consistent variable coding and standardized data capture across different electronic medical record systems impact predictive performance and clinical utility in real-world ED environments. Such validation efforts would enable health authorities to establish evidence-based URV benchmarks and quality metrics that support systematic ED performance monitoring and resource allocation strategies across healthcare networks. The epidemiological insights derived from consistent URV prediction patterns could inform targeted public health interventions and healthcare system preparedness planning.

Third, integration of this prediction model into ED triage protocols will enable real-time risk assessment to support clinical decision-making and intervention targeting. Future implementations could incorporate free-text clinical data including patient chief complaints and physician narrative documentation to develop multimodal architectures combining TabNet’s structured data processing with LLM analysis of unstructured clinical text, potentially capturing semantic nuances in symptom descriptions and clinical reasoning. Achieving this integration requires future research to prioritize comprehensive data capture systems that preserve both structured variables and free-text narratives, alongside lightweight computational architectures and standardized deployment frameworks that facilitate seamless integration with hospital information systems while maintaining the distinct roles of each AI component within the clinical workflow.

## Conclusion

This study demonstrates that TabNet exhibits superior performance compared to conventional ML approaches in predicting ED URVs. Through systematic comparison of multiple AI architectures, DL frameworks designed for tabular data analysis were found to provide distinct advantages in capturing URV risk patterns. The identified predictive factors establish a robust foundation for implementing AI-enabled decision support systems in emergency care settings, where early risk identification may enhance both resource utilization and patient outcomes.

## Data Availability

The original contributions presented in the study are included in the article/Supplementary material, further inquiries can be directed to the corresponding author.
