# Supplementary material for: Performance comparison of artificial intelligence models in predicting 72-h emergency department unscheduled return visits
Source: Front Public Health. 2025 Dec 19;13:1609206. doi: 10.3389/fpubh.2025.1609206 (PMC12757422; doi:10.3389/fpubh.2025.1609206)
Supplement: Supplementary file 1 [file Supplementary_file_1.docx]

**Supplementary**

**Table S1**. Variables, definitions, and timing of availability for 72-h ED URV analysis

| Category | Variable Name | Description | Timing of Availability |
| --- | --- | --- | --- |
| Basic Information | Patient ID | Unique identifier for each patient | Registration |
|  | Age | Actual age (years) |  |
|  | Gender | Male: 0, Female: 1 |  |
| Common Medical History | Hypertension | No: 0, Yes: 1 | Initial assessment |
|  | Diabetes | No: 0, Yes: 1 |  |
|  | Cardiovascular Disease | No: 0, Yes: 1 |  |
|  | Hyperlipidemia or Fatty Liver | No: 0, Yes: 1 |  |
|  | Stroke | No: 0, Yes: 1 |  |
| Initial Triage Parameters | Body Temperature | Measured in degrees Celsius (℃), | Triage assessment |
|  | Systolic Blood Pressure | Measured in mmHg |  |
|  | Diastolic Blood Pressure | Measured in mmHg |  |
|  | Heart Rate | Measured in beats per minute |  |
|  | Blood Oxygen Saturation | Measured in % |  |
|  | Triage Level | P1: 1, P2: 2, P3: 3, P4: 4 |  |
| Behavioral Characteristics | Activity Ability | Independent: 0, Accompanied: 1 | Triage assessment |
|  | ED Visits Last Month | Number of ED visits in the past month |  |
| Diagnostic Procedures | CT Examination | No: 0, Yes: 1 | Initial clinical assessment |
|  | Ultrasound Examination | No: 0, Yes: 1 |  |
|  | ECG Examination | No: 0, Yes: 1 |  |
|  | MRI Examination | No: 0, Yes: 1 |  |
| Initial Diagnosis | Respiratory System Disease | No: 0, Yes: 1 | Initial clinical assessment |
|  | Cardiovascular System Disease | No: 0, Yes: 1 |  |
|  | Digestive System Disease | No: 0, Yes: 1 |  |
|  | Nervous System Disease | No: 0, Yes: 1 |  |
|  | Urinary System Disease | No: 0, Yes: 1 |  |
| Visit and Provider Characteristics | Date of Initial Visit | Date of initial ED visit | Initial clinical assessment |
|  | Physician Shift | Morning: 1, Afternoon: 2, Night: 3 |  |
|  | Day Type | Weekday: 0, Holiday: 1 |  |
|  | Physician Seniority | Attending Physician: 1, Associate Senior Physician: 2, Senior Physician: 3 |  |

Note: Triage levels are based on the Patient Acuity Category (PAC) scale, where P1 indicates life-threatening conditions requiring immediate attention, P2 represents serious conditions needing early intervention, P3 refers to minor emergencies, and P4 denotes non-urgent cases.

**Table S2**. Optimal hyperparameter configurations of AI models for URV risk prediction following Bayesian optimization

| Model | Parameter | Values |
| --- | --- | --- |
| LR | C | 0.093 |
|  | Penalty | 12 |
|  | Solver | saga |
|  | Tolerance (tol) | 1e-4 |
|  | Random State | 42 |
| RF | Number of Estimators (n_estimators) | 263 |
|  | Max Features (max_features) | sqrt |
|  | Max Depth (max_depth) | 10 |
|  | Min Samples Split (min_samples_split) | 5 |
|  | Min Samples Leaf (min_samples_leaf) | 2 |
| SVM | C | 0.604 |
|  | Kernel | rbf |
|  | Gamma | scale |
|  | Probability | True |
|  | Random State | 42 |
| XGBoost | Number of Estimators (n_estimators) | 258 |
|  | Learning Rate (learning_rate) | 0.089 |
|  | Max Depth (max_depth) | 4 |
|  | Colsample Bytree | 0.654 |
|  | Gamma | 0 |
|  | Subsample | 0.887 |
| TabNet | Decision Dimension (n_d) | 18 |
|  | Attention Dimension (n_a) | 20 |
|  | Decision Steps (n_steps) | 3 |
|  | Gamma | 1.5 |
|  | Seed | 42 |
|  | Learning Rate (lr) | 0.01 |

C: Regularization parameter, Gamma: Minimum loss reduction, sqrt: Square root, rbf: Radial basis function, saga: Stochastic average gradient with acceleration

**Table S3**. Calibration performance metrics

| Model | Brier score | Intercept | Slope | ECE |
| --- | --- | --- | --- | --- |
| TabNet | 0.008  (0.007, 0.085) | -0.03  (-0.09, 0.07) | 1.08  (0.91, 1.19) | 0.079  (0.063, 0.144) |
| RF | 0.004  (0.002, 0.048) | 0.03  (-0.02, 0.11) | 0.96  (0.86, 1.04) | 0.066  (0.047, 0.132) |
| SVM | 0.023  (0.009, 0.057) | -0.04  (-0.10, 0.06) | 1.07  (0.89, 1.15) | 0.099  (0.077, 0.160) |
| XGBoost | 0.020  (0.014, 0.053) | -0.16  (-0.20, -0.07) | 1.44  (1.29, 1.54) | 0.114  (0.091, 0.171) |
| LR | 0.051  (0.046, 0.068) | 0.23 (0.12, 0.35) | 0.58  (0.09, 0.99) | 0.211  (0.155, 0.291) |

ECE: Expected calibration error. Values in parentheses represent 95% CI.

**Table S4.** Net benefit analysis for predictive models across clinically relevant decision thresholds

| Threshold | TabNet | RF | SVM | XGBoost | LR |
| --- | --- | --- | --- | --- | --- |
| 10% | 0.117  (0.114, 0.121) | 0.120  (0.117, 0.124) | 0.116  (0.112, 0.120) | 0.104  (0.100, 0.108) | 0.086  (0.082, 0.090) |
| 15% | 0.095  (0.091, 0.099) | 0.092  (0.088, 0.095) | 0.093  (0.089, 0.097) | 0.076  (0.072, 0.079) | 0.063  (0.059, 0.066) |
| 20% | 0.072  (0.068, 0.076) | 0.065  (0.061, 0.069) | 0.065  (0.062, 0.070) | 0.046  (0.042, 0.050) | 0.043  (0.040, 0.047) |
| 25% | 0.050  (0.046, 0.054) | 0.038  (0.035, 0.042) | 0.037  (0.033, 0.041) | 0.014  (0.010, 0.019) | 0.023  (0.018, 0.027) |
| 30% | 0.028  (0.024, 0.032) | 0.011  (0.006, 0.015) | 0.009  (0.004, 0.013) | -0.021  (-0.025, -0.017) | -0.001  (-0.005, 0.004) |

Values in parentheses represent 95% CI.


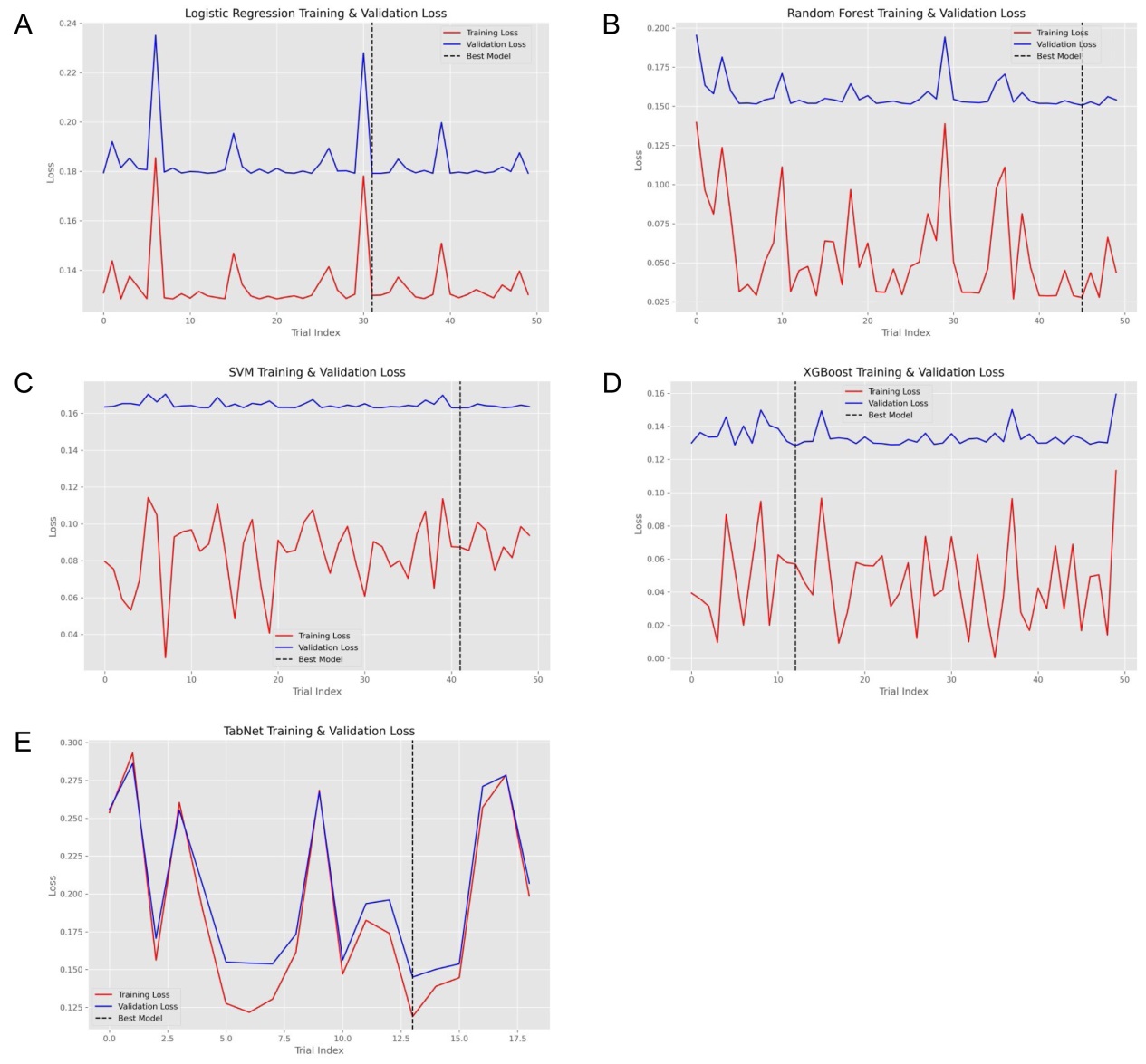


**Figure S1**. Training and validation loss curves during hyperparameter optimization trials for different models. (A) LR, (B) RF, (C) SVM, (D) XGBoost, and (E) TabNet. The red and blue lines represent training and validation loss trajectories, respectively. The vertical dashed lines indicate the trial with optimal hyperparameter configuration that achieved the minimum validation loss.
